# Human induced pluripotent stem cell-based platform for modeling cardiac ischemia

**DOI:** 10.1038/s41598-021-83740-w

**Published:** 2021-02-18

**Authors:** Martta Häkli, Joose Kreutzer, Antti-Juhana Mäki, Hannu Välimäki, Henna Lappi, Heini Huhtala, Pasi Kallio, Katriina Aalto-Setälä, Mari Pekkanen-Mattila

**Affiliations:** 1 Heart Group, Faculty of Medicine and Health Technology, Tampere University, Arvo Ylpön katu 34, 33520 Tampere, Finland; 2 Micro- and Nanosystems Research Group, Faculty of Medicine and Health Technology, Tampere University, Tampere, Finland; 3 Faculty of Social Sciences, Tampere University, Tampere, Finland

**Keywords:** Induced pluripotent stem cells, Cardiovascular models

## Abstract

Ischemic heart disease is a major cause of death worldwide, and the only available therapy to salvage the tissue is reperfusion, which can initially cause further damage. Many therapeutics that have been promising in animal models have failed in human trials. Thus, functional human based cardiac ischemia models are required. In this study, a human induced pluripotent stem cell derived-cardiomyocyte (hiPSC-CM)-based platform for modeling ischemia–reperfusion was developed utilizing a system enabling precise control over oxygen concentration and real-time monitoring of the oxygen dynamics as well as iPS-CM functionality. In addition, morphology and expression of hypoxia-related genes and proteins were evaluated as hiPSC-CM response to 8 or 24 h hypoxia and 24 h reoxygenation. During hypoxia, initial decrease in hiPSC-CM beating frequency was observed, after which the CMs adapted to the conditions and the beating frequency gradually increased already before reoxygenation. During reoxygenation, the beating frequency typically first surpassed the baseline before settling down to the values close the baseline. Furthermore, slowing on the field potential propagation throughout the hiPSC-CM sheet as well as increase in depolarization time and decrease in overall field potential duration were observed during hypoxia. These changes were reversed during reoxygenation. Disorganization of sarcomere structures was observed after hypoxia and reoxygenation, supported by decrease in the expression of sarcomeric proteins. Furthermore, increase in the expression of gene encoding glucose transporter 1 was observed. These findings indicate, that despite their immature phenotype, hiPSC-CMs can be utilized in modeling ischemia–reperfusion injury.

## Introduction

Ischemic heart disease (IHD) is the most common cardiovascular disease and a major cause of death worldwide^1^. In IHD, blood flow to myocardium is reduced or blocked leading to oxygen and nutrient deprivation, and accumulation of metabolic waste in the tissue. This causes damage and death to the cells in the myocardium, including cardiomyocytes (CMs) that are responsible for contraction of heart^2^. While a lot of research resources has been invested to the study of the disease, reperfusion, i.e. restoring blood flow to the ischemic tissue, is currently the only available therapy to reduce damage to the ischemic area in addition to prevention of arrhythmias by antiarrhythmic medication. However, reperfusion itself causes further damage to the tissue. Animal experiments have provided promising results for medical interventions at the time of reperfusion, but they have failed in human clinical trials^3^. As the difference between species is considered to be one reason behind the failure^4,5^, it is important to establish functional human based models to evaluate and develop new therapies.

Human induced pluripotent stem cells (hiPSCs) can be endlessly produced and efficiently differentiated into cardiomyocytes (hiPSC-CMs) enabling development of human-based cell models for the research of cardiac diseases and therapies^6^. However, hiPSC-CMs are developmentally immature and structurally, functionally and metabolically resemble more fetal than adult CMs^7^. Especially, the metabolic immaturity affects the use of the hiPSC-CMs in ischemia modeling, as their metabolism relies on glucose making them more resistant to hypoxia and reperfusion. Adult CMs mainly utilize fatty acid oxidation in energy production and thus are more vulnerable to oxidative stress than fetal CMs^8–10^.

Despite their drawbacks, hiPSC-CM based ischemia–reperfusion models have lately emerged^8–23^ and these models have been reported to recapitulate the typical cardiac ischemia responses in the cells, such as increased cell death^8–10^ and disruption of the sarcomere structure^9,10^. Furthermore, changes in the functionality and electrophysiology have been observed, including decrease in hiPSC-CM beating frequency^11,12,15,16,24^, contractility, calcium overload and arrhythmias^14^. In these models, hypoxic conditions have been induced to the hiPSC-CMs by multiple methods, such as by using hypoxic gas^8–10,13–15,24,25^ or causing oxidative stress via peroxide treatment^16^. In addition, cell culture media composition has been modified to better recapitulate the ischemic conditions in several studies. The modifications include removal of glucose^23^ or serum^11^, or both^8–10,13,14,22^, as well as acidosis to better mimic the physiological ischemic event^8–10,13,22^.

In this study, we present a hiPSC-CM based platform for modeling cardiac ischemia–reperfusion. The base of the platform is an OxyGenie mini-incubator^26^ combined with a microelectrode array (MEA) and a luminescence-based oxygen sensor^12,24^. The system allows precise control over oxygen concentration, real-time monitoring of the cardiomyocyte functionality as well as the measurement of oxygen level in the cell culture area during hypoxia and reoxygenation without disturbing or interrupting the experiment. The main objective of the present study was develop a platform to assess the functional, structural and molecular responses of hiPSC-CMs to ischemia and reperfusion. The hiPSC-CMs were combined with an extensive set of analysis methods including a microelectrode array and oxygen measurement. In addition, the platform is compatible with collecting and analyzing samples for immunocytochemistry, qPCR and western blot. As the studies on the electrophysiology and functionality of hiPSC-CMs under hypoxia and reoxygenation are very limited in their number, this study strongly contributes to the hiPSC-CM based ischemia–reperfusion modeling.

## Results

### hiPSC-CM functionality during hypoxia and reoxygenation

Functionality of the hiPSC-CMs was studied with MEA to evaluate changes in the beating characteristics of the hiPSC-CMs during hypoxia and reoxygenation. For beating frequency, signals from 41 electrodes were analyzed (4–6 electrodes from 9 samples, 3 parallel samples from 3 differentiation batches). The luminescence-based oxygen measurement was incorporated to one sample to evaluate the oxygen dynamics of the platform. It was observed that after initiation of hypoxia or reoxygenation, the oxygen level in the culture stabilized within four hours. Clear and repeatable changes were observed in the beating frequency of the hiPSC-CMs during hypoxia and reoxygenation, which together with the observed oxygen dynamics were used to divide hypoxia and reoxygenation to periods for statistical analysis.

**Figure 1:**
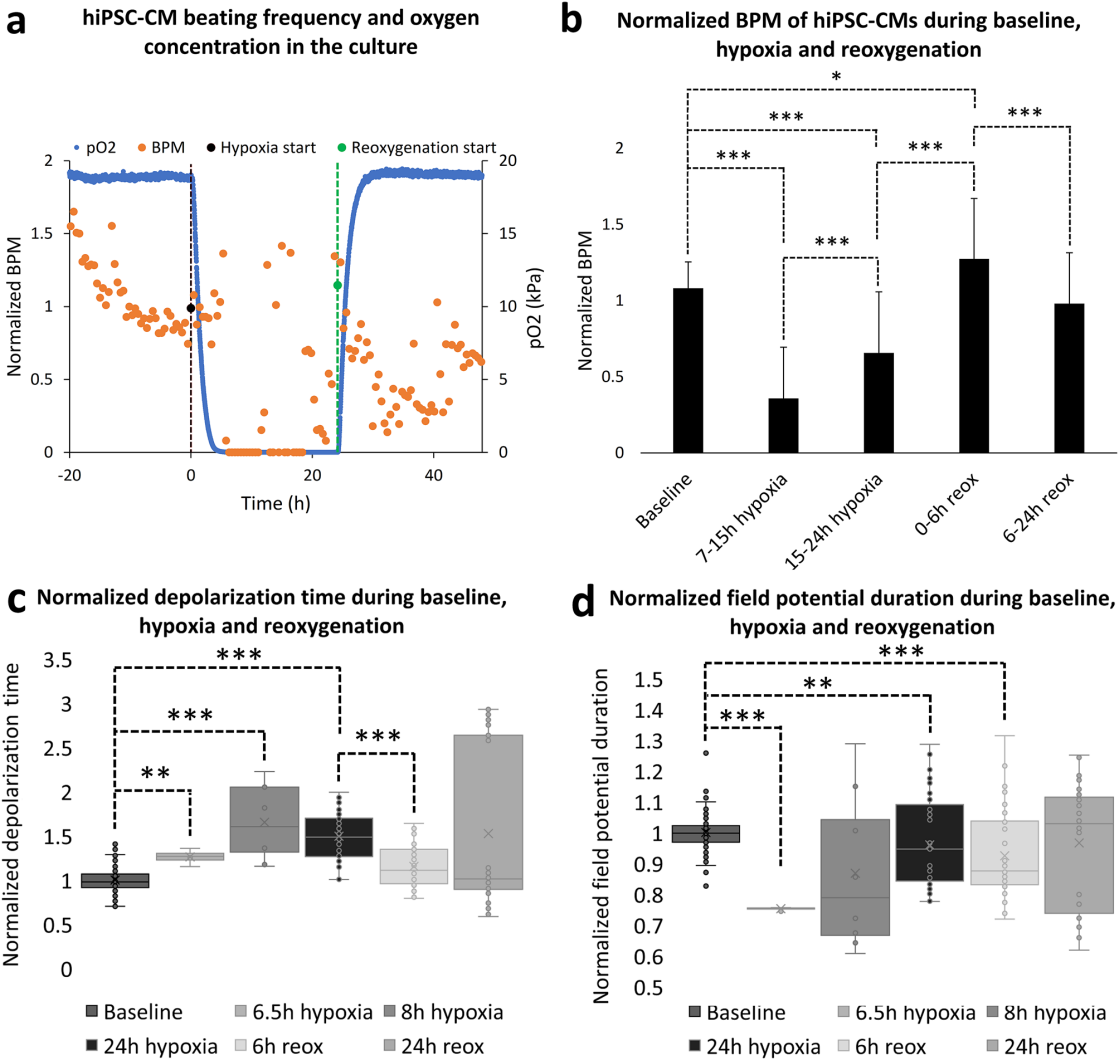
(**a**) Representative measurement of the partial pressure of oxygen and normalized beating frequency from a single experiment. Black line marks the initiation of hypoxia, after which the oxygen level of the culture starts to decrease and reaches 0 kPa approximately within 4 h. Green line marks the initiation of reoxygenation, after which the oxygen level starts to increase and reaches 19 kPa approximately within 4 h of reoxygenation. The beating frequency of the hiPSC-CMs clearly decreases during hypoxia and increases after reoxygenation. (**b**) The mean normalized beating frequency extracted from MEA recordings for hiPSC-CMs during baseline measurement, 7–15 h hypoxia, 15–24 h hypoxia, 0–6 h reoxygenation and 6–24 h reoxygenation presented as mean + standard deviation (n_electrodes_ = 41). Normalization is done to each electrode separately with regard to median baseline value, due to the variation observed in the beating frequency between different electrodes during baseline measurements. The beating frequency starts to decrease after hypoxia is initiated and was observed to be on its lowest during 7–15 h of hypoxia. The beating frequency started to recover already before reoxygenation was initiated and was statistically significantly greater during 15–24 h hypoxia compared to 7–15 h hypoxia. Furthermore, overcompensation in the beating frequency was observed during 0–6 h of reoxygenation, when the mean normalized beating frequency exceeded the baseline. After 6 h reoxygenation, the beating frequency returned close to the baseline level. (**c**) Normalized depolarization time of hiPSC-CMs at baseline, 6.5 h, 8 h and 24 h hypoxia as well as 6 h and 24 h reoxygenation. Depolarization time increased during hypoxia and returned close to the baseline level during reoxygenation. (**d**) Normalized field potential duration (FPD) of hiPSC-CMs at baseline, 6.5 h, 8 h and 24 h hypoxia as well as 6 h and 24 h reoxygenation. FPD decreased during hypoxia and returned close to the baseline level during reoxygenation *p < 0.05, **p < 0.01, ***p < 0.001.

For beating frequency analysis, hypoxia was divided into two periods, 7–15 h hypoxia and 15–24 h hypoxia, as during 7–15 h hypoxia the oxygen level had already stabilized and the beating frequency of the hiPSC-CMs was lowest, while it gradually started to increase during 15–24 h of hypoxia. Reoxygenation was divided into two groups as well; 0–6 h reoxygenation to observe the immediate increase of the beating frequency during the increase of the pO_2_, and 6–24 h reoxygenation, during which the beating of the hiPSC-CMs stabilized. Figure 1a presents a representative measurement of pO_2_ and a normalized beating frequency from a single electrode of one MEA measurement, while Fig. S1 presents examples of a normalized beating rate from other experiments. Fig. S2 presents a representative MEA signal during different time points of hypoxia and reoxygenation and Fig. S3 presents the post-calibration of the oxygen measurement.

Beating frequency (beats per minute, BPM) was normalized for each electrode to a median baseline BPM value so that the results are comparable despite the differences in the baseline BPM. Median baseline BPM value was chosen for normalization instead of mean so that individual time points that deviate much from the baseline do not affect the normalization. The baseline was determined from measurements of 20 h before initiation of hypoxia and it was relatively stable throughout the time period. Mean of the normalized beating frequency of all the chosen electrodes at baseline was 1.081 ± 0.173 and started to decrease after hypoxia started. During 7–15 h hypoxia, the normalized beating frequency was 0.358 ± 0.336 (p = 2.4*10^–8^ compared to baseline) and during 15–24 h hypoxia, it increased to 0.656 ± 0.402 (p = 8.8*10^–7^ compared to baseline and p = 2.6*10^–5^ compared to 7–15 h hypoxia). During 0–6 h reoxygenation, the normalized beating frequency increased to 1.274 ± 0.396 (p = 0.015 compared to baseline and p = 2.8*10^–8^ compared to 15–24 h hypoxia) and then decreased close to the baseline level during 6–24 h reoxygenation, to 0.981 ± 0.333 (p = 3.0*10^–6^ compared to 0–6 h reoxygenation). Figure 1b presents the mean normalized beating frequency at the baseline, 7–15 h hypoxia, 15–24 h hypoxia, 0–6 h reoxygenation and 6–24 h reoxygenation.

Depolarization time and field potential duration (FPD) were evaluated from three samples at two timepoints of baseline measurement (n_spikes_ = 19 for both time points for all three samples), as well as from one sample after 6.5 h (n_spikes_ = 4) and from two samples after 8 h hypoxia (n_spikes_ = 4 for both samples), depending on whether beating had stopped already at 8 h. Depolarization time and FPD were also evaluated from the three samples after 24 h of hypoxia (n_spikes_ = 13 for all three samples), and 6 h (n_spikes_ = 17 for all three samples) and 24 h (n_spikes_ = 14 for all three samples) of reoxygenation. Depolarization time was determined as the time from the first peak to the second peak, whereas FPD was determined as the time from the first peak to the flat peak as shown in Fig. S4. Both parameters were normalized for each sample to the mean of their own baseline values. As a response to hypoxia, depolarization time increased while the total FPD decreased, whereas during reoxygenation, both values returned close to the baseline level again, as demonstrated in Fig. 1c,d. Compared to baseline, the mean depolarization time increased 1.26 ± 0.09 -fold at 6.5 h hypoxia (p = 0.0044), 1.65 ± 0.43 -fold at 8 h hypoxia (p = 1.3*10^–5^) and 1.49 ± 0.27 -fold at 24 h hypoxia (p = 8.67*10^–17^). At 6 h reoxygenation, the increase was 1.15 ± 0.24 -fold (p = 0.00033 compared to baseline, p = 2.64*10^–7^ compared to 24 h hypoxia) whereas at 24 h reoxygenation it was 1.52 ± 0.92 -fold (not significant) compared to baseline. On the other hand, compared to baseline, the total field potential duration decreased to 0.75 ± 0.01 at 6.5 h hypoxia (p = 0.0007), 0.87 ± 0.25 at 8 h hypoxia (not significant), and then increased to 0.96 ± 0.15 at 24 h hypoxia (p = 0.0085), 0.92 ± 0.14 at 6 h reoxygenation (p = 1.0*10^–5^), and 0.97 ± 0.19 at 24 h reoxygenation (not significant).

**Figure 2:**
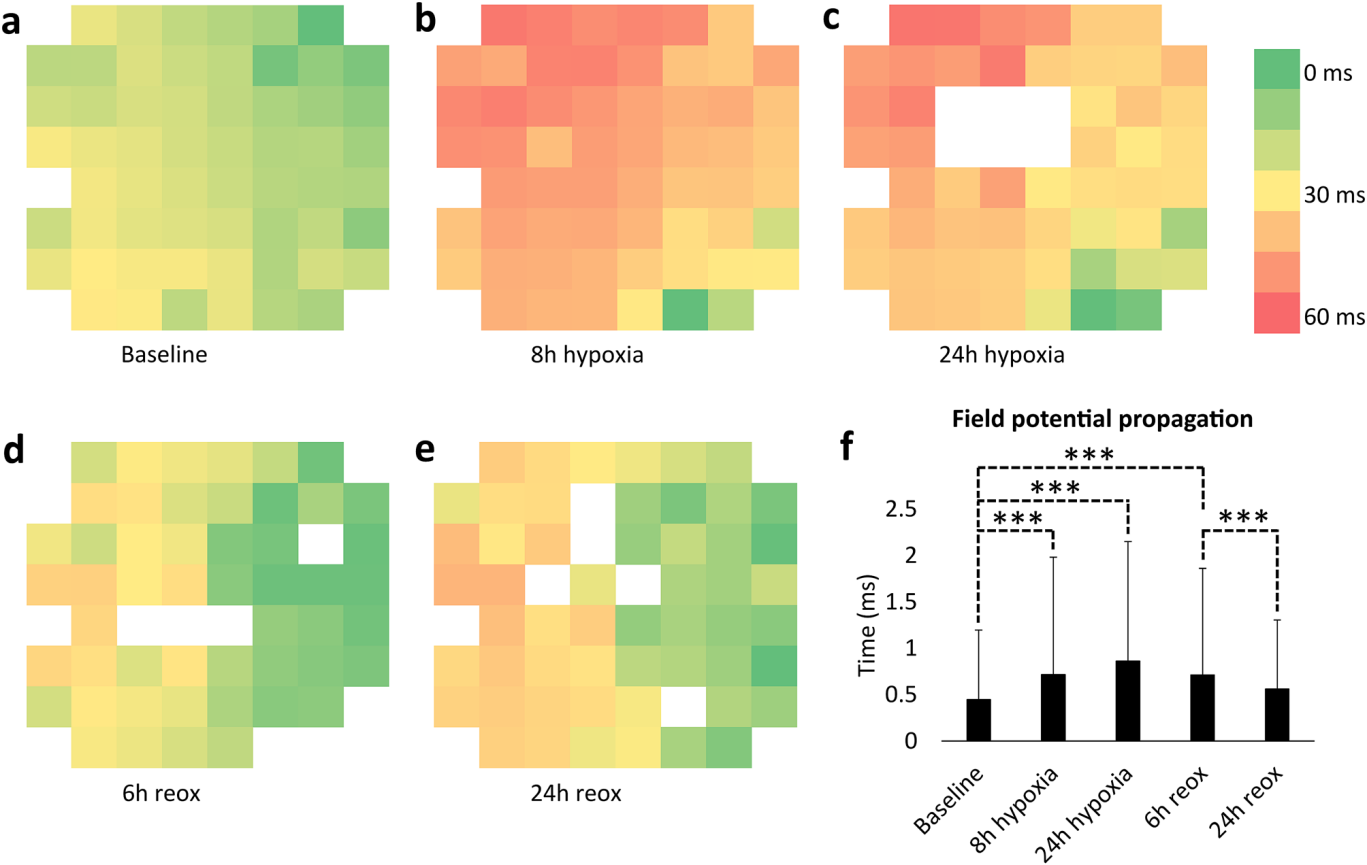
(**a**–**e**) Field potential propagation over hiPSC-CM sheet on microelectrode array at baseline before hypoxia, at 8 h and 24 h hypoxia and at 6 h and 24 h reoxygenation. The heatmaps clearly indicate slowing of the field potential conduction during hypoxia, but the conduction velocity is restored during reoxygenation. f) Field potential conduction time from electrode to electrode at baseline, 8 h and 24 h hypoxia, and 6 h and 24 h reoxygenation (mean + standard deviation). Conduction time increased during hypoxia compared to baseline, whereas it was restored close to baseline level during reoxygenation. ***p < 0.001.

Signal propagation was evaluated at two baseline timepoints, as well as after 8 h and 24 h hypoxia and 6 h and 24 h reoxygenation from all signals of all electrodes of two samples that were showing field potential signals. The conduction velocity was observed to slow down during hypoxia and increase close to the baseline level during reoxygenation, as shown in Fig. 2a–f. At baseline, the mean duration of field potential propagation from one electrode to the adjacent (in x or y direction) was observed to be 0.45 ± 0.75 ms, whereas it was 0.72 ± 1.26 at 8 h hypoxia (p = 6.4*10^–18^), 0.86 ± 1.29 ms at 24 h hypoxia (p = 5.2*10^–35^). At 6 h reoxygenation, the time was observed to be 0.71 ± 1.15 ms (p = 1.1*10^–40^ compared to baseline) and at 24 h reoxygenation it was 0.56 ± 0.74 ms (p = 3.8*10^–5^ compared to 6 h reoxygenation).

### hiPSC-CM morphology, sarcomere structure and nucleus size

**Figure 3:**
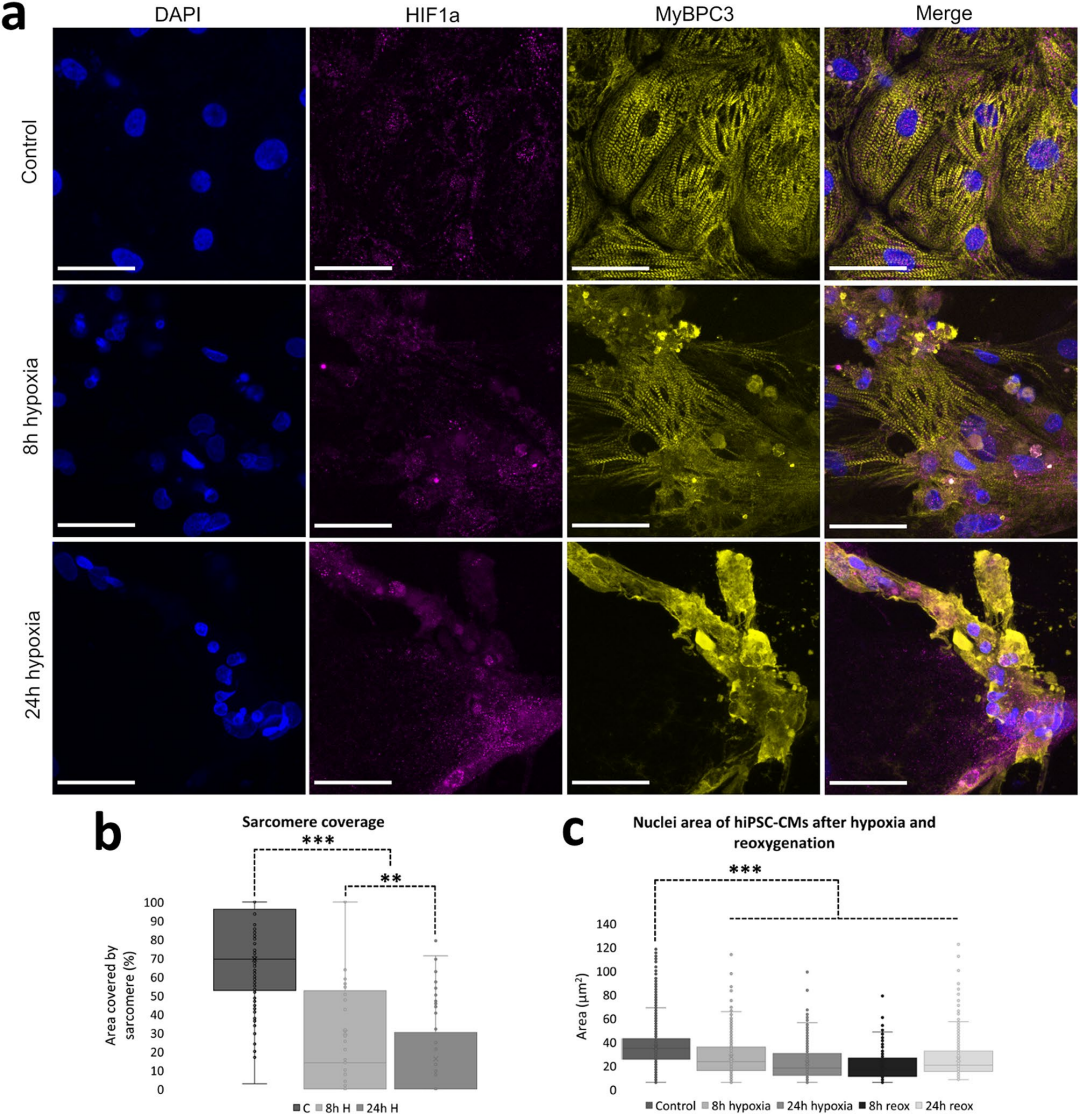
(**a**) The structure, sarcomere expression and nucleus size changes during hypoxia. Sarcomeres (MyBPC3, yellow) are clearly visible and the cells are spread and properly attached to the plate in control (n_images_ = 124) samples, whereas the cell structure seems to deteriorate after 8 h (n_images_ = 47) and 24 h (n_images_ = 50) hypoxia. Furthermore, the nucleus (DAPI, blue) size seems to decrease. On the other hand, no significant changes are seen in the expression of hypoxia marker HIF1α (magneta). (**b**) Sarcomere coverage was quantified from the fluorescent images and calculated as the ratio between the area of visible sarcomeres to the total cell area and is expressed as percentage. The data are presented as mean + standard deviation. The expression of clear and visible sarcomeres decreases as the length of the hypoxia increases. (**c**) Area of the nuclei in control (n_nuclei_ = 3632), 8 h hypoxia (n_nuclei_ = 685), 24 h hypoxia (n_nuclei_ = 833), 8 h hypoxia-reoxygenation (n_nuclei_ = 329) and 24 h hypoxia-reoxygenation (n_nuclei_ = 782) samples presented as mean + standard deviation. The area of the nuclei decreases with hypoxia and reoxygenation compared to control. **p < 0.01; ***p < 0.001.

The morphology, sarcomere structure, nucleus size and HIF1α expression of the hiPSC-CMs was studied with immunocytochemistry. After exposure to hypoxia or hypoxia-reoxygenation, hiPSC-CMs were immunolabeled against MyBPC3 and HIF1α and cell nuclei were visualized with DAPI (Fig. 3a). 6 hypoxia and control samples (2 parallel samples from 3 differentiation batches) from both 8 and 24 h experiments as well as 4 hypoxia-reoxygenation samples (2 parallel samples from 2 differentiation batches) from both 8 and 24 h experiments were stained and imaged. Qualitative analysis revealed changes in the cell morphology, expression of sarcomeres and the size of the nuclei, which were then quantitated and determined statistically significant. However, the expression of HIF1α was not found to increase in any of the used time points.

Quantitative analysis on sarcomere expression and nuclei size of the cells revealed statistically significant differences in these parameters between hypoxia or hypoxia-reoxygenation and control samples (Fig. 3b,c). Sarcomere coverage was analyzed from 124 images of control samples, 47 images of 8 h hypoxia samples and 50 images of 24 h hypoxia samples. The mean coverage of clear and distinguishable sarcomeres of the total area of the MyBPC3 staining in control samples was 69.4 ± 24.0%, whereas for 8 and 24 h hypoxia samples it was 30.1 ± 36.0% (p = 4.3*10^–10^) and 16.1 ± 24.8% (p = 6.1*10^–18^), respectively. Nucleus area was analyzed from the fluorescent microscopy images (n_8h hypoxia-reox_ = 18, n_24h hypoxia-reox_ = 21) using CellProfiler. The mean nucleus area was 35.7 ± 16.4 µm^2^ for control samples (n_nuclei_ = 3632), whereas for 8 and 24 h hypoxia samples it was respectively 27.2 ± 15.2 µm^2^ (n_nuclei_ = 685, p = 8.9*10^–43^) and 22.3 ± 13.5 µm^2^ (n_nuclei_ = 833, p = 1.5*10^–113^), and 8 and 24 h hypoxia-reoxygenation samples it was respectively 19.8 ± 11.1 µm^2^ (n_nuclei_ = 329, p = 5.1*10^–74^) and 25.4 ± 14.6 µm^2^ (n_nuclei_ = 782, p = 8.5*10^–74^).

### Western blot of HIF1α, MyBPC3 and Troponin T

**Figure 4:**
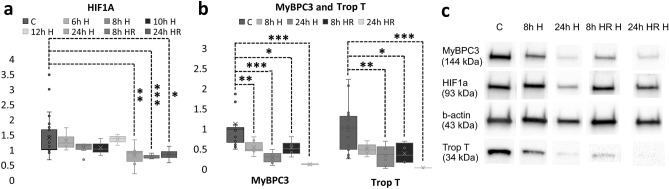
(**a**) HIF1α expression in control (n = 26) samples and in 6 h (n = 4), 8 h (n = 6), 10 h (n = 3), 12 h (n = 3) and 24 h (n = 6) hypoxia or 8 h (n = 4) and 24 h (n = 4) hypoxia-reoxygenation time points from western blot analysis presented as mean + standard deviation. During the first 12 h of hypoxia, there seems to be no significant changes in the expression of the protein, although in 6 h and 12 h samples there is a slight increase. On the other hand, after 24 h of hypoxia, as well as after 8 h hypoxia-reoxygenation there is a statistically significant decrease in the HIF1α expression. (**b**) Expression of MyBPC3 and Troponin T decreases during 8 h (n = 6) and 24 h (n = 6) hypoxia, and 8 h (n = 4) and 24 h (n = 4) hypoxia-reoxygenation compared to control (n = 20) samples. The expression is especially low in 24 h hypoxia-reoxygenation samples but has decreased also in other hypoxia samples. (**c**) Examples of the western blot bands. *p < 0.05; **p < 0.01; ***p < 0.001.

Western blot was used to quantitate the expression of HIF1α, MyBPC3 and Troponin T after hypoxia or hypoxia-reoxygenation. After 6 h (n = 4, 1 differentiation batch), 8 h (n = 6, 2 parallel samples from 3 differentiation batches), 10 h (n = 3, 1 differentiation batch) and 12 h (n = 3, 1 differentiation batch) hypoxia, the HIF1α expression remained close to control (n = 26, 4–5 parallel samples from 6 differentiation batches), but after 24 h hypoxia as well as 8 and 24 h hypoxia-reoxygenation (n = 4 for both time points, 2 parallel samples from 2 differentiation batches), there was a statistically significant decrease in the HIF1α expression (Fig. 4a). The mean expression of HIF1α in control samples was 1.39 ± 0.68, whereas for 6, 8, 10, 12 and 24 h hypoxia samples it was respectively 1.29 ± 0.32, 1.05 ± 0.20, 1.06 ± 0.28, 1.34 ± 0.18 and 0.80 ± 0.39 (p = 0.006). For 8 and 24 h hypoxia-reoxygenation samples, the expression of HIF1α was 0.77 ± 0.08 (p = 0.001) and 0.84 ± 0.23 (p = 0.031), respectively.

There were statistically significant decreases in the expressions of MyBPC3 and Troponin T after hypoxia and hypoxia-reoxygenation (Fig. 4b). The mean MyBPC3 expression in the control samples was 1.11 ± 0.76, whereas for 8 and 24 h hypoxia samples it was 0.53 ± 0.18 (p = 0.006) and 0.28 ± 0.32 (p = 0.000009), respectively. For 8 and 24 h hypoxia-reoxygenation samples the expression of MyBCP3 was 0.52 ± 0.22 (p = 0.023) and 0.11 ± 0.02 (p = 0.0002), respectively. As for Troponin T, the mean expression in the control samples was 0.96 ± 0.55. For 8 and 24 h hypoxia samples, the expression was respectively 0.47 ± 0.14 and 0.32 ± 0.27 (p = 0.007), while for 8 and 24 h hypoxia-reoxygenation samples the expression was 0.37 ± 0.26 (p = 0.037) and 0 (p = 0.0002), respectively. In 24 h hypoxia-reoxygenation samples, the Troponin T band did not become visible with exposure times suitable for control samples. Examples of the western blot bands are presented in Fig. 4c, whereas full length blots of the examples are presented in Supplementary Figures S5–S6.

### Gene expression of hiPSC-CMs

**Figure 5:**
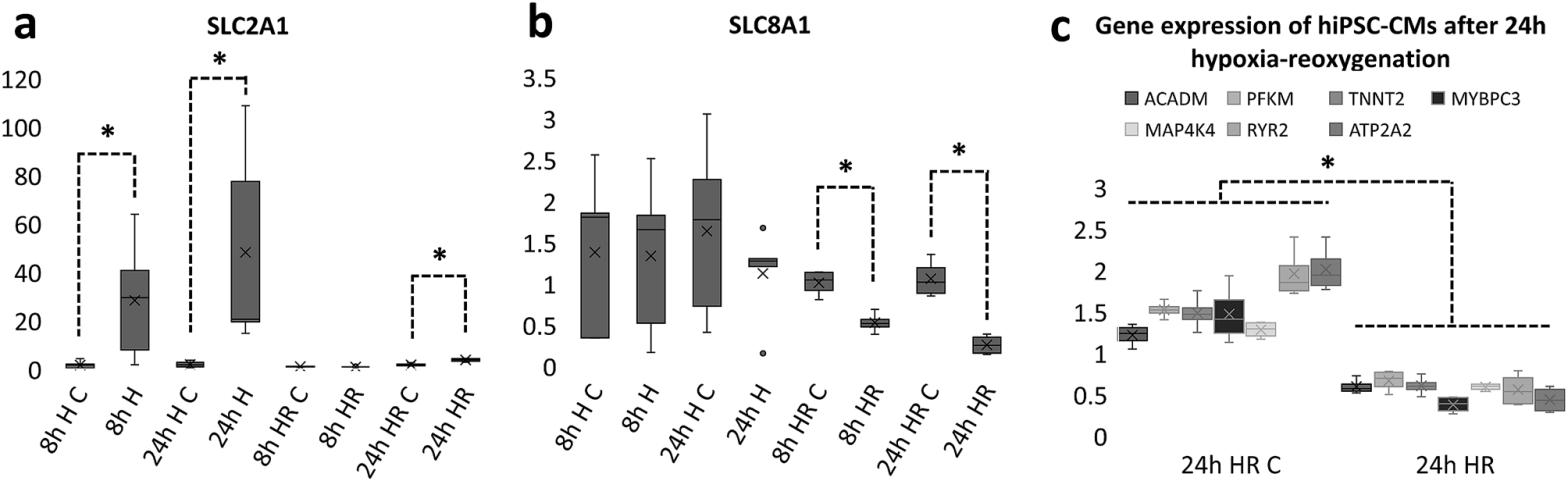
(**a**) SLC2A1 expression in hiPSC-CMs after 8 and 24 h hypoxia in control (C, n_8h_ = 6, n_24h_ = 5) and hypoxia (H, n_8h_ = 5, n_24h_ = 6) samples as well as 8 and 24 h hypoxia-reoxygenation (HR) in control (C, n_8h_ = 4, n_24h_ = 4) and hypoxia (H, n_8h_ = 4, n_24h_ = 4) samples from qPCR analysis presented as mean + standard deviation. SLC2A1 encodes glucose transporter 1 and is related to glucose uptake of the cells. As can be seen, SLC2A1 expression increases during hypoxia, and decreases during reoxygenation. However, after 24 h hypoxia-reoxygenation, the expression levels are still higher than in control. (**b**) SLC8A1 expression in hiPSC-CMs after 8 and 24 h hypoxia as well as 8 and 24 h hypoxia-reoxygenation. SLC8A1 encodes sodium/calcium exchanger and is related to calcium handling of hiPSC-CMs. As can be seen from the figure, the expression decreases after 8 and 24 h hypoxia-reoxygenation, but does not change significantly during hypoxia only. (**c**) Expression of several genes after 24 h hypoxia-reoxygenation. The expression of metabolic genes (ACADM and PFKM), sarcomeric genes (TNNT2 and MYBPC3), calcium handling genes (RYR2 and ATP2A2) and hypoxia marker MAP4K4 are all decreasing when compared to control. *p < 0.05.

Expression of several genes related to glucose and fatty acid metabolism, calcium handling, sarcomeric proteins, hypoxia and apoptosis was analyzed from samples from 8 h (n_hypoxia_ = 6, n_control_ = 5, 1–2 parallel samples from 3 differentiation batches) and 24 h (n_hypoxia_ = 5, n_control_ = 6, 1–2 parallel samples from 3 differentiation batches) hypoxia and 8 and 24 h hypoxia-reoxygenation experiments (n_hypoxia_ = 4, n_control_ = 4 for both time points, 2 parallel samples from 2 differentiation batches) with qPCR. There were no statistically significant differences in the expression of most genes after 8 or 24 h hypoxia. However, after 24 h hypoxia-reoxygenation, there was a statistically significant decrease in several genes. A statistically significant increase was observed in the expression of SLC2A1 encoding glucose transporter 1 in all but 8 h hypoxia-reoxygenation samples (Fig. 5a). For 8 h control and hypoxia samples, the mean expressions of SLC2A1 were 1.70 ± 1.65 and 28.32 ± 24.13 (p = 0.03), respectively. For 24 h control and hypoxia samples, the expressions were 1.88 ± 1.33 and 48.22 ± 42.47 (p = 0.004), respectively. For 8 h control and hypoxia-reoxygenation samples, the expressions were 1.01 ± 0.16 and 0.85 ± 0.13, respectively. For 24 h control and hypoxia-reoxygenation samples, the expressions were 1.77 ± 0.68 and 3.76 ± 0.77 (p = 0.029), respectively.

In 8 and 24 h hypoxia-reoxygenation samples, there was also a statistically significant decrease in the expression of SLC8A1 encoding sodium/calcium exchanger 1 related to calcium handling of cardiomyocytes (Fig. 5b). Slight but not statistically significant decrease was also seen in 24 h hypoxia samples. For 8 h control and hypoxia samples, the expressions were 1.38 ± 0.99 and 1.34 ± 0.95, while for 24 h control and hypoxia samples they were 1.64 ± 1.06 and 1.12 ± 0.57. For 8 h control and hypoxia-reoxygenation samples, the expressions were 1.01 ± 0.16 and 0.53 ± 0.12 (p = 0.029), respectively. For 24 h control and hypoxia-reoxygenation samples, the expressions were 1.06 ± 0.23 and 0.26 ± 0.12 (p = 0.029), respectively.

In 24 h hypoxia-reoxygenation samples, there was also statistically significant decrease in the expression of ACADM (c = 1.22 ± 0.13, 24 h HR = 0.60 ± 0.09, p = 0.029), PFKM (c = 1.52 ± 0.10, 24 h HR = 0.67 ± 0.13, p = 0.029), TNNT2 (c = 1.48 ± 0.21, 24 h HR = 0.60 ± 0.11, p = 0.029), MYBPC3 (c = 1.47 ± 0.35, 24 h HR = 0.38 ± 0.10, p = 0.029), MAP4K4 (c = 1.28 ± 0.10, 24 h HR = 0.59 ± 0.04, p = 0.029), RYR2 (c = 1.96 ± 0.31, 24 h HR = 0.56 ± 0.20, = 0.029) and ATP2A2 (c = 2.01 ± 0.28, 24 h HR = 0.44 ± 0.16, p = 0.029) (Fig. 5c).

## Discussion

In the present study, a human based cardiac ischemia–reperfusion model which enables precise control and real time monitoring of oxygen combined with simultaneous measurement of the electrophysiological parameters of hiPS-CMs is presented. Contrary to our previous studies, in which the similar system was used with hiPS-CM aggregates^11,12,24^, here the hiPS-CMs were sorted and re-plated as a monolayer on top of the MEA-plates providing more homogenous iPS-CM population and enabling a detailed assessment of the structural characteristics of the cells. The more homogenous iPS-CM population also enables more precise qPCR analysis of cardiac markers while the samples contain approximately similar percentage of cardiomyocytes.

According to the results, hypoxia as well as reoxygenation alters the function of hiPSC-CMs significantly. The lowest beating frequency in the hiPSC-CMs was observed during 7–15 h hypoxia, whereas during 15–24 h hypoxia, the beating frequency gradually increased, suggesting that the hiPSC-CMs adapt to the low oxygen environment. During 0–6 h reoxygenation, the mean normalized beating frequency further increased and surpassed the baseline level but returned close to the baseline during 6–24 h reoxygenation. However, it is noteworthy that majority of the hiPSC-CMs retained their functionality after an initial drop in the beating frequency, during the late phase of hypoxia.

The decrease in cardiomyocyte beating frequency during ischemic stress has been earlier reported in primary CMs from rat^27–29^, in perfused whole mouse heart^30^. We and others have also reported the decrease with pluripotent stem cell derived-CMs ^11,12,24,31^, as well as the increase in the beating frequency upon reoxygenation^11,12,24,27,29–31^.

The decrease in the beating rate might occur due to decrease in the ATP content in the CMs^27,29^ and due to the conduction block affecting the propagation of the action potential from pacemaker cells to other cells^31^. Reoxygenation is thought to reverse these changes, restoring the beating frequency close to the baseline^27,29,31^. However, to our knowledge, this is the first time that an increase in the CM beating frequency already during a late phase of hypoxia has been reported. The reason behind the hiPSC-CM adaptation is likely balancing of the ATP supply and demand^32^, although the mechanisms for achieving the balance can only be speculated.

The immature metabolic phenotype of the hiPSC-CMs can be partially responsible for the observed adaptation of the cells and increase of the beating frequency with prolonged hypoxia. hiPSC-CMs are known to be more resistant to hypoxia and reoxygenation because of the enhanced ability of immature CMs to increase glycolytic flux^33,34^. In the current study, increase in the expression of SLC2A1 encoding glucose transporter 1 was observed after 8 and 24 h hypoxia. During hypoxia, the CM metabolism relies on glycolysis, which is a relatively inefficient way to produce energy requiring increase in glucose uptake and expression of glucose transporters^35^. However, the expression of PFKM gene encoding muscle type ATP-dependent 6-phosphofructokinase responsible for the first committing step of glycolysis^35^ did not increase after 8 or 24 h hypoxia. Furthermore, the medium used in the experiment did not contain either glucose or serum since presence of glucose attenuates the adverse effects of hypoxia^36^. Thus, there must be also another mechanism for the hiPSC-CM functional adaptation.

In addition to changes in the beating frequency of the hiPSC-CMs as a response to hypoxia and reoxygenation, changes in depolarization time and field potential duration were investigated. Hypoxia increased the depolarization time, whereas it decreased the overall FPD. During reoxygenation, both the depolarization time and the FPD returned close to the baseline level. These findings are in line with other studies regarding prolongation of CM action potential depolarization time as well as shortening of the overall action potential duration during hypoxia and reoxygenation^37^ and indicate that the hiPSC-CMs in the model function as expected.

Changes in the conduction velocity over the hiPSC-CM sheets were also investigated. During hypoxia, the signal propagation speed slowed down, and it took longer for the action potential to travel across the cell sheet. This could be due to the conduction blocks that are known to occur in ischemic conditions^38^, which can prevent the action potential from traveling the shortest route through the cell sheet. The slowing was observed to be reversible, as the conduction velocity returned close the baseline level during reoxygenation. These findings are in agreement with King et al. (2013), where delayed signal propagation has also been observed as response to ischemia^38^.

There were no significant changes in the expression of genes related to calcium handling and contractility of the hiPSC-CMs after 8 or 24 h hypoxia. However, post-transcriptional changes in the proteins or changes in their activity due to hypoxia could in part explain the increase in the beating frequency. Electrophysiological alterations can include decrease in sodium and potassium currents, thus reducing the ATP demand of Na^+^/K^+^ ATPase. Decrease in calcium current could similarly reduce the ATP demand of Ca^2+^ ATPases as well as Na^+^/K^+^ ATPases. Reduced potassium current would also lead to increased resting membrane potential making the CMs more excitable, although the membrane potential must remain below the activation voltage of voltage-gated sodium channels^32^. The initial decrease in the hiPSC-CM beating frequency can thus later be accompanied by electrophysiological changes sufficiently reducing the ATP demand for the gradual increase in beating frequency during the late phase of hypoxia.

A peak in the HIF1α expression was not found in this study. However, it is possible that the peak occurs already earlier than the chosen time points, or that the peak is subtle and thus difficult to observe. Since there was some variation in the expression between the samples from the same time points, it is possible that the subtle increase in the expression of HIF1α was masked by the variation of the individual samples. It is also possible, that oxygen independent HIF1α degradation pathways are activated^39^. Furthermore, nuclear translocation of HIF1α was not seen in the immunocytochemical staining, although it is known to play a crucial role in activation of hypoxia pathways^40^.

Differences in hiPSC-CM morphology and sarcomere structure were observed after hypoxia and hypoxia-reoxygenation, which were supported by western blot analysis of the structural proteins. The morphological and structural changes in the hiPSC-CMs included partial detachment from the culture plate as well as disruption of the visible sarcomere structures. Furthermore, the area of the nuclei in the hiPSC-CMs decreased during hypoxia and hypoxia-reoxygenation. However, cell death was not observed (data not shown). The structural disintegration and the decrease in the nucleus size are associated with apoptotic cell death^41,42^. However, the difference in the nucleus area could also partially be due to detachment of the cells, because in attached cells the nucleus is flat against the culture plate thus increasing the area in images, while in detached cells the nucleus has a more 3D spherical shape, which is not conveyed from 2D images. Furthermore, western blot analysis showed no band for cleaved caspase-3 associated with apoptosis.

The cellular damage in the hiPSC-CMs could also be indication of necrotic cell death, which is known to be the primary mechanism of cell death in ischemia-reperfusion^41,42^. The decrease in MyBPC3 and Troponin T protein expression further supports the structural changes seen in the hiPSC-CMs. The decrease in Troponin T expression could be due to the release of Troponin T from the cells to the surroundings, although it was not measured in this study. However, Troponin T release from injured myocardium is a clinically used marker for myocardial infarction^43^. Furthermore, necrotic cell death is associated with spillage of cellular contents into the extracellular space.

One limitation in this study was the immature phenotype of the hiPSC-CMs. As already mentioned, differences in hiPSC-CM metabolism make these cells more resistant to hypoxia-reoxygenation injury. Another limitation is the relatively constant pH throughout the hypoxia and reoxygenation treatments. Due to the large volume of medium compared to the cell mass, the pH of the culture did not change significantly during the hypoxia or reoxygenation. However, in heart, ischemia is accompanied by acidosis, a decrease in intracellular pH, which is known to affect the CM response to ischemia–reperfusion in primary animal CM models^28^ and hiPSC-CMs^8,10^. The third limitation is the slow speed in increase and decrease of the oxygen level in the culture, which gives the cells time to adjust to the conditions, whereas acute ischemic events are typically sudden in the clinical situation. Furthermore, electrical activity was recorded only in intervals, which could cause missing of potentially interesting events.

In summary, we conclude that hiPS-CM model presented in this study can be used in modeling cardiac ischemia–reperfusion injury. With the iPS-CM based platform, we are able to monitor the temporal changes in cardiomyocyte function as well as electrophysiological parameters during different stages of ischemia–reperfusion event. There were significant differences in the beating frequency, depolarization time and field potential duration as well as conduction velocity. In addition, hypoxia altered the cell morphology and caused changes in the expression of structural proteins. With the presented hiPS-CM based platform, cardiac ischemia–reperfusion can be modeled and these parameters can be utilized when evaluating the effects of putative ischemia drugs and treatments in the future.

## Materials and methods

### Cardiomyocyte differentiation and cell culture

UTA.04602.WT hiPSC line^44^ was used in the experiments. hiPSCs were cultured on mouse embryonic fibroblast feeder cells (Applied StemCells, Inc) in KSR medium (KnockOut DMEM (Gibco) containing 10% KnockOut Serum Replacement (Gibco), 1% MEM NEAA (Gibco), 1% GlutaMAX (Gibco), 0.2% β-mercaptoethanol (Gibco) and 0.5% Penicillin/streptomycin (Lonza)). Embryoid body differentiation modified from Karakikes and coworkers^45^ and Lian and coworkers^46^ was used to differentiate the hiPSCs into cardiomyocytes (full protocol in Supplementary Information).

### Magnetic activated cell sorting

Magnetic activated cell sorting (MACS) was performed on day 20 to enrich the cardiomyocyte concentration in the culture. MultiTissue Dissociation Kit (Miltenyi Biotec) was used according to manufacturer’s instructions as described earlier^47^. PSC-Derived Cardiomyocyte Isolation Kit, human (Miltenyi Biotec) was used to separate the cardiomyocytes from other cell types according to manufacturer’s instructions^47^. The CMs were suspended to 20% EB medium (KnockOut DMEM containing 20% FBS (Gibco), 1% MEM NEAA, 1% GlutaMAX and 0.5% Penicillin/streptomycin) and seeded to 1-well chambers on glass or MEA coated with 0.1% gelatin as cell sheets (density ~ 93,000 cells/cm^2^). The cells were cultured for 7–9 days before starting the hypoxia experiments. Half of the culture medium was exchanged three times a week.

### Hypoxia and hypoxia-reoxygenation

**Figure 6:**
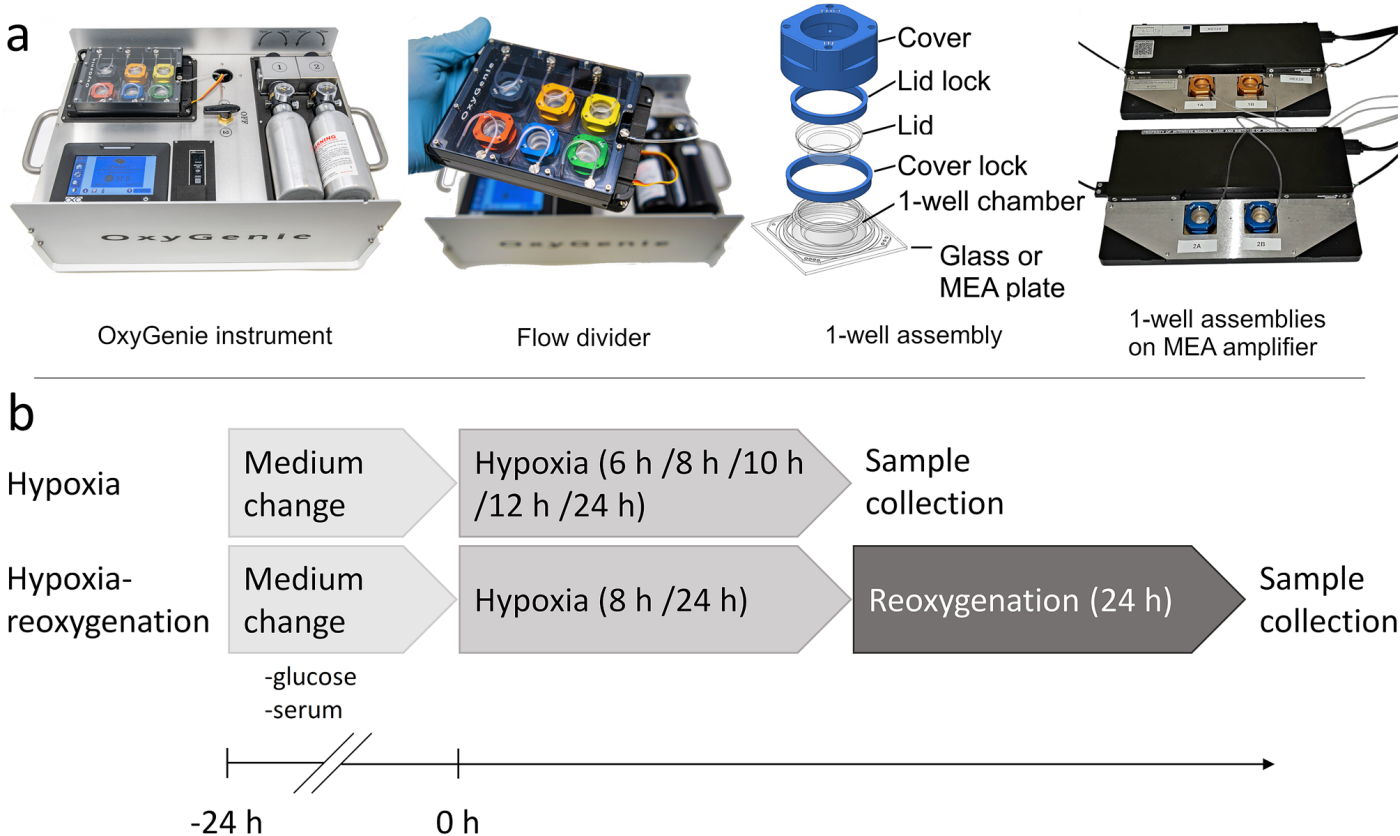
(**a**) Portable cell culture instrument include battery operated temperature controller and heat plate, two gas cylinders and flow divider allowing six individual 1-well assemblies. 1-well assembly includes 1-well chamber on plate (glass or MEA), the lid and the lid lock to seal the cell culture and to avoid evaporation, the cover and the cover lock to create and maintain the gas environment around gas permeable 1-well chamber. 1-well assembly on MEA plate can be fitted also to MCS signal amplifier and thus use it for long-term recordings outside the incubator. (**b**) Hypoxia and hypoxia-reoxygenation protocol.

Hypoxia and reoxygenation were performed using OxyGenie mini-incubator (Baker, USA), which is based on our previous studies^26,48,49^. The portable cell culture incubator includes a battery-operated temperature controller with a heat plate, two prefilled and replaceable gas cylinders and a flow divider to hold six individual 1-well chamber assemblies on a glass plate (Fig. 6a). The 1-well assembly on an MEA plate fits to a MultiChannel Systems signal amplifier allowing long-term recordings outside an incubator. Portability of the OxyGenie and the individual 1-well chambers enable sample preparation and collection one at a time, minimizing the cell culture exposure to ambient air.

Day before exposing cells to hypoxia, serum- and glucose-free EB medium (glucose-free DMEM (Gibco) containing 1% MEM NEAA, 1% GlutaMAX and 0.5% Penicillin/streptomycin) was changed. On the following day, the samples were loaded into pre-warmed (37 °C) OxyGenie mini-incubator and hypoxia was initiated using 0% O_2_ and 5% CO_2_ (hypoxic) gas. The used time periods included 6, 8, 10, 12 and 24 h. Control samples were cultured in serum- and glucose-free EB medium in a standard 5% CO_2_ incubator without the lids and covers for the same time periods.

Hypoxia-reoxygenation experiments were performed similarly to the hypoxia experiments, but after 8 or 24 h hypoxia, the gas was exchanged to 19% O_2_ and 5% CO_2_ (normoxic) gas for 24 h. The control cells were kept in the 1-well chambers on the glass inside the incubator for the whole experiment. Samples were collected after the hypoxia or hypoxia-reoxygenation one by one to minimize the exposure to ambient air. pH was measured from cell culture medium during sample collection using Sentron SI600 pH meter with Sentron MicroFET pH probe.

With MEA plates, hypoxia-reoxygenation was performed so that first the samples were connected to normoxic gas to measure a baseline overnight, after which, hypoxia was initiated using the hypoxic gas for 24 h. After the hypoxia, reoxygenation was performed by connecting the samples again to the normoxic gas for another 24 h. Hypoxia and hypoxia-reoxygenation protocols are presented in Fig. 6b.

### Microelectrode array

Microelectrode array (MEA) plates (60MEA200/30iR-Ti, 8 × 8) were ordered from MultiChannel Systems MCS GmbH (Reutlingen, Germany and PDMS 1-well chambers attached to the MEA were manufactured inhouse^50^. MEA measurements were performed using MultiChannel Experimenter (version 2.14.0.19346, Multi Channel Systems, GmbH, Reutlingen, Germany). Sampling frequency was set to 25 kHz and the baseline was recorded for 1 min every hour for 20 h. During hypoxia and reoxygenation, MEA signals were recorded for 1 min every 30 min. The data were converted from MSRD into HDF5 format using MultiChannel DataManager (version 1.12.0.20014, Multi Channel Systems, GmbH, Reutlingen, Germany). The HDF5 files were analyzed with MATLAB (version R2018B, MathWorks, Inc., Natick, MA, USA) using scripts developed inhouse for beating frequency, depolarization time and field potential duration. Multi Channel Analyzer (version 2.14.0.19346, Multi Channel Systems, GmbH, Reutlingen, Germany) was used to detect peaks from the recorded data. The analyzed data was converted into ASCII files using Multi Channel DataManager and these files were analyzed for field potential propagation.

### Oxygen measurement

The partial pressure of oxygen (pO_2_) was measured from one sample to verify that the oxygen dynamics in the cell culture were as expected. In the verification experiment, a luminescence-based sensor together with a highly biocompatible sensing material, developed by Välimäki and coworkers^24^ was used to monitor the oxygen level in the culture. The pO_2_ was measured once a minute during the entire experiment, while MEA signals were recorded for 1 min every 30 min throughout the experiment at 20 kHz sampling frequency. After the experiment was finished, the cells were removed from the MEA plate and the oxygen measurement was calibrated using the same MEA.

### Immunocytochemistry

Immunocytochemistry (full protocol in Supplementary Information) was performed right after hypoxia or hypoxia-reoxygenation for 8 and 24 h hypoxia, and 8 and 24 h hypoxia-reoxygenation samples. Mouse anti-MyBPC3 (1:500; Santa Cruz; sc-166081) and rabbit anti-HIF1α (1:1000; Invitrogen; 700505) were used as primary antibodies and donkey anti-mouse Alexa Fluor 568 and donkey anti-rabbit Alexa Fluor 488 (1:800; Thermo Fisher Scientific) as secondary antibodies. Fluorescence was visualized with Zeiss LSM 800 Laser Scanning Confocal Microscope using Zeiss EC Plan-Neofluar 40x/0.75, WD 0.71 mm (Air) objective and 2 channel spectral detection with high-sensitivity PMT detector.

### Analysis of hiPSC-CM sarcomere coverage

The area of visible sarcomeres and the total cell area was analyzed from the MyBPC3 channel of the fluorescent microscopy images using Paint.net (version 4.1.5, DotPDN, LLC, USA) and ImageJ^51^. The wand tool in Paint.net was used to determine the total area of the cells covering the image and the area covered by sarcomeres. ImageJ was then used to calculate the areas. Sarcomere coverage was calculated as the ratio between the area of visible sarcomeres and the total cell area and is expressed as percentage.

### Analysis of hiPSC-CM nuclei area

The nucleus area was determined using Cell Profiler^52^. IdentifyPrimaryObjects was used to identify the nuclei in the DAPI channel of the fluorescent images. The minimum and maximum object size values were set as 50 and 250, respectively. Objects outside the set minimum and maximum as well as objects touching borders were discarded. Threshold strategy was set as global and the thresholding method used was two class Otsu. Shape was set for the method to distinguish clumped objects and MeasureObjectSizeShape was used to determine the nuclei area from the objects identified in the IdentifyPrimaryObjects.

### Western blot

Protein samples were collected in 2 × Laemmli buffer (Bio-Rad) containing 5% β-mercaptoethanol (Sigma) and run in 4–20% Mini PROTEAN TGX Precast Protein Gel with 10 50 µl wells (Bio-Rad). The proteins were blotted from the gel to PVDF membrane using Trans-Blot Turbo Transfer System (Bio-Rad) and Trans-Blot Turbo RTA Mini PVDF Transfer Kit (Bio-Rad). Mouse anti-β-actin (1:1000; Santa Cruz; sc-47778), rabbit anti-HIF1α (1:1000), rabbit anti-cleaved caspase-3 (1:500; Abcam; ab32042), mouse anti-MyBPC3 (1:500) and mouse anti-Troponin T (1:1000; Abcam; ab33589) were used as primary antibodies and horseradish peroxidase-conjugated anti-mouse IgG (1:3000; Santa Cruz; sc-516102) and anti-rabbit IgG (1:2000; Dako; P0217) as secondary antibodies (full protocol in Supplementary Information). The protein-antibody complexes were detected using Amersham ECL Prime Western Blotting Detection Reagent (GE Healthcare Life Sciences) and ChemiDoc MP Imaging System (Bio-Rad) was used for imaging. The images were analyzed using Image Lab Software (Bio-Rad).

### RNA extraction, reverse transcription and qPCR

Samples for qPCR from hypoxia and hypoxia-reoxygenation experiments were collected in QIAzol Lysis Reagent (Qiagen) and RNA was extracted using miRNeasy Mini Kit (Qiagen) following manufacturer’s instructions. RNA quality was ensured using. Reverse transcription (RT) was performed using High-Capacity cDNA Reverse Transcription Kit (Applied Biosystems), following manufacturer’s instructions. Shortly, 2X RT Master Mix was prepared based on instructions of the kit and 10 µl of 2X RT Master Mix was mixed with 10 µl of sample. Samples were run in thermal cycler with following protocol: 10 min at 25 °C, 120 min at 37 °C, 5 min at 85 °C and at 4 °C until samples were stored at − 20 °C.

qPCR for mRNA samples was performed using TaqMan Gene Expression Master Mix (Applied Biosystems) following manufacturer’s instructions. TaqMan 20 × Assays for TNNT2, MYBPC3, ACADM, ACAA1, SLC2A1, PFKM, RYR2, ATP2A2, SLC8A1, MAP4K4, HIF1A, CASP3, GAPDH, EEF1A1 and TBP were used, details of the assays are presented in Table 1. Master mixes were prepared as instructed in the kit. Three technical replicates were used for each sample with each gene. 1 µl of diluted cDNA (diluted 2:1 to Milli-Q water) was pipetted into 9 µl of master mix (total reaction volume 10 µl). Plates were run in ABI7300 thermal cycler (Applied Biosystems) with following protocol: 2 min at 50 °C, 10 min at 95 °C and 40 cycles of 15 s at 95 °C and 1 min at 60 °C.

**Table 1:** TaqMan 20 × Assays used in qPCR.

Gene	Description	Function	Assay ID
ACADM	Acyl-CoA Dehydrogenase Medium Chain	Fatty acid metabolism	Hs00936584_m1
ACAA1	Acetyl-CoA acyltransferase 1	Fatty acid metabolism	Hs01576070_m1
PFKM	Phosphofructokinase, muscle	Glycolysis metabolism	Hs01075411_m1
SLC2A1	Solute carrier family 2, member 1/GLUT-1	Glycolysis metabolism	Hs00892681_m1
TNNT2	Cardiac type troponin T2	Sarcomeric	Hs00165960_m1
MYBPC3	Myosin binding protein C3, cardiac	Sarcomeric	Hs00165232_m1
RYR2	Ryanodine receptor 2, cardiac	Calcium handling	Hs00892883_m1
ATP2A2	ATPase, calcium transporting, cardiac muscle, slow twitch 2/ SERCA2a	Calcium handling	Hs00544877_m1
SLC8A1	Solute carrier family 8, member 1/NCX1	Calcium handling	Hs01062258_m1
HIF1A	Hypoxia inducible factor 1 alpha	Hypoxia marker	Hs00153153_m1
MAP4K4	Mitogen-activated protein kinase kinase kinase kinase 4	Hypoxia marker	Hs00377415_m1
CASP3	Caspase-3	Apoptosis	Hs00234387_m1
GAPDH	Glyceraldehyde-3-phosphate dehydrogenase	Housekeeping	Hs02758991_g1
TBP	TATA-box binding protein	Housekeeping	Hs00427620_m1
EEF1A1; EE +	Eukaryotic translation elongation factor 1 alpha 1	Housekeeping	Hs00265885_g1

### Statistical analysis

Statistical analyses were performed with IMB SPSS Statistics for Windows (version 25.0, IMB Corp., Armonk, NY, USA). Independent samples Mann–Whitney U test was used to test the statistical significance between control and hypoxia or hypoxia-reoxygenation groups for data extracted from qPCR, western blot, immunostaining, depolarization time and field potential duration and field potential propagation, while related-samples Wilcoxon Signed Rank Test was used for beating frequency. p < 0.05 was considered as statistically significant. The data are presented as mean ± standard deviation.

## Supplementary Information

Supplementary Information

## Data Availability

All data are available from the authors by request.
