# Supplementary material for: Human induced pluripotent stem cell-based platform for modeling cardiac ischemia
Source: Sci Rep. 2021 Feb 18;11:4153. doi: 10.1038/s41598-021-83740-w (PMC7893031; doi:10.1038/s41598-021-83740-w)
Supplement: Supplementary file 1 — Supplementary Information [file 41598_2021_83740_MOESM1_ESM.pdf]

Title: Human induced pluripotent stem cell-based system for modeling cardiac ischemia

Authors

Martta Häkli[1], Joose Kreutzer[2], Antti-Juhana Mäki[2], Hannu Välimäki[2], Henna Lappi[1], Heini Huhtala[3], Pasi Kallio[2], Katriina Aalto-Setälä[1] and Mari Pekkanen-Mattila[1]

[1] Heart Group, Faculty of Medicine and Health Technology, Tampere University, Tampere, Finland

[2] Micro- and Nanosystems Research Group, Faculty of Medicine and Health Technology, Tampere University, Tampere, Finland

[3] Faculty of Social Sciences, Tampere University, Tampere, Finland

Corresponding author: Martta Häkli, MSc. Tech., Arvo Ylpön katu 34, 33520, Tampere, +358 50 360 0987, martta.hakli@tuni.fi, https://orcid.org/0000-0003-3617-6959

Supplementary Information

Supplementary Methods

Differentiation

On day 0, hiPSCs were detached using Versene (Gibco), suspended to mTeSR1 (Stemcell Technologies) medium containing 0.5% Penicillin/streptomycin and 5 μM Blebbistatin (Sigma) and transferred to ultra-low attachment 6-well plates to form embryoid bodies. On day 1, all medium was exchanged to differentiation medium (RPMI 1640 + GlutaMAX supplement medium (Gibco) containing 1% B-27 Minus Insulin (50X) (Gibco) and 0.5% Penicillin/streptomycin) supplemented with 5 μg/ml ascorbic acid (Sigma), 10 ng/ml BMP4 (R&D Systems) and 25 ng/ml activin A (Peprotech). On day 3, all medium was exchanged to differentiation medium supplemented with 5 μg/ml ascorbic acid. On day 4, all medium was exchanged to differentiation medium supplemented with 2,5 μM IWP-4 (Tocris). On day 7, all medium was exchanged to differentiation medium. From day 9 on, half of the medium was exchanged three times a week. From day 11 forward, B-27 Supplement (50X), serum free (Gibco) was used. On day 13, the cells were plated down to 12-well plates coated with 0.1% porcine skin gelatin (Sigma-Aldrich) diluted in milliQ-grade water.

Immunocytochemistry

The samples were fixed in 4% paraformaldehyde for 20 min, blocked in 10% normal donkey serum (Biowest) solution for 45 min and stained against mouse anti-MyBPC3 (1:500; Santa Cruz; sc-166081) and rabbit anti-HIF1α (1:1000; Invitrogen; 700505) at 4 °C overnight. Donkey anti-mouse Alexa Fluor 568 and donkey anti-rabbit Alexa Fluor 488 (1:800; Thermo Fisher Scientific) were used as secondary antibodies (1 h incubation). The cell nuclei were stained using Vectashield mounting medium with DAPI (Vector Laboratories).

Western blot

After protein transfer from gel to PVDF membranes, blocking was done with 3% bovine serum albumin (BSA) (Sigma-Aldrich) in 0.1% Tween20 (Sigma) in tris-buffered saline (TBS) for 3 hours at RT. After blocking, the membranes were incubated in primary antibody dilutions mouse anti-β-actin (1:1000; Santa Cruz; sc-47778), rabbit anti-HIF1α (1:1000), rabbit anti-cleaved caspase-3 (1:500; Abcam; ab32042), mouse anti-MyBPC3 (1:500) and mouse anti-Troponin T (1:1000; Abcam; ab33589) overnight at 4 °C. All primary and

secondary antibodies were diluted in 3% BSA in 0.1% TBS-Tween. After primary antibody incubation, the membranes were washed in 0.5% TBS-Tween, 0.1% TBS-Tween and 0.05% TBS-Tween. Horseradish peroxidase-conjugated anti-mouse IgG (1:3000; Santa Cruz; sc-516102) and anti-rabbit IgG (1:2000; Dako; P0217) were diluted in 3% BSA in 0.1% TBS-Tween and membranes were incubated in the solutions at RT for 1 hour. The membranes were washed as after primary antibody incubation and protein-antibody complexes were detected using Amersham ECL Prime Western Blotting Detection Reagent (GE Healthcare Life Sciences). ChemiDoc MP Imaging System (Bio-Rad) was used for imaging, after which the antibodies were stripped in stripping buffer (0.705% β-mercaptoethanol, 2% SDS and 0.03125 M Tris in Milli-Q water) for 30 min at 56 °C. The membranes were washed after which new blocking was started.

Supplementary Figures

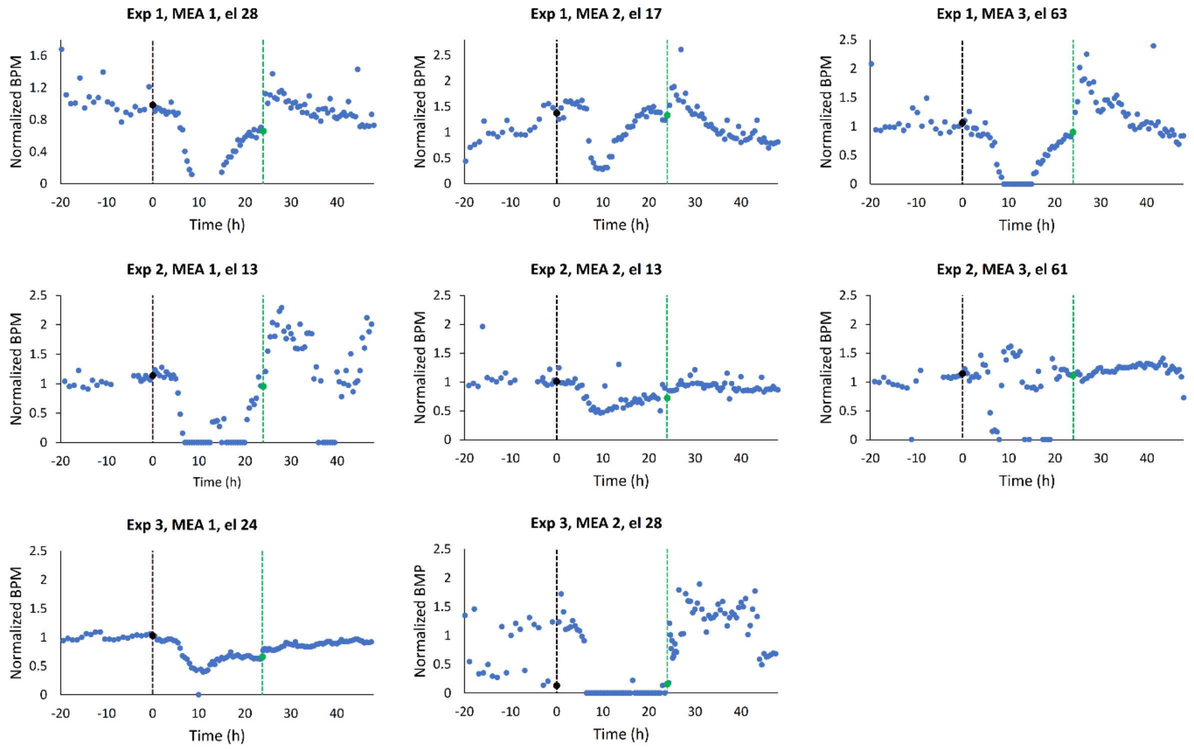

Supplementary Figure S 1. Examples of normalized beating frequencies of the hiPSC-CMs from several separate experiments. Black line marks hypoxia initiation and green line reoxygenation initiation. The trend in the beating frequency is very similar in each experiment. There is a clear decrease in the beating frequency during hypoxia, but the CMs seem to recover before reoxygenation starts. After reoxygenation, overcompensation can be seen in several samples, before the beating frequency returns close to baseline after approximately 6 hours of reoxygenation.

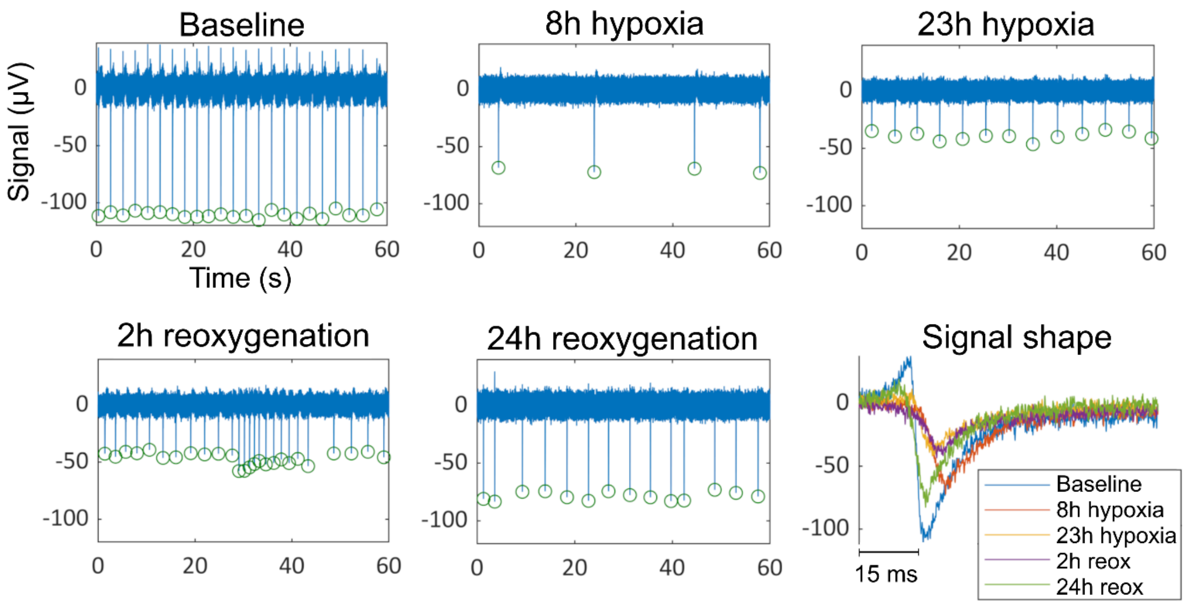

Supplementary Figure S 2. Examples of recorded MEA signals during baseline, 8 h hypoxia, 23 h hypoxia, 2 h reoxygenation and 24 h reoxygenation. Green circles mark counted beats. Examplary signal shape of one beat from each time point is also presented in upper right corner. Althoug variation in the beat-to-beat interval was observed, no clear arrhythmias were identified from the functionality data.

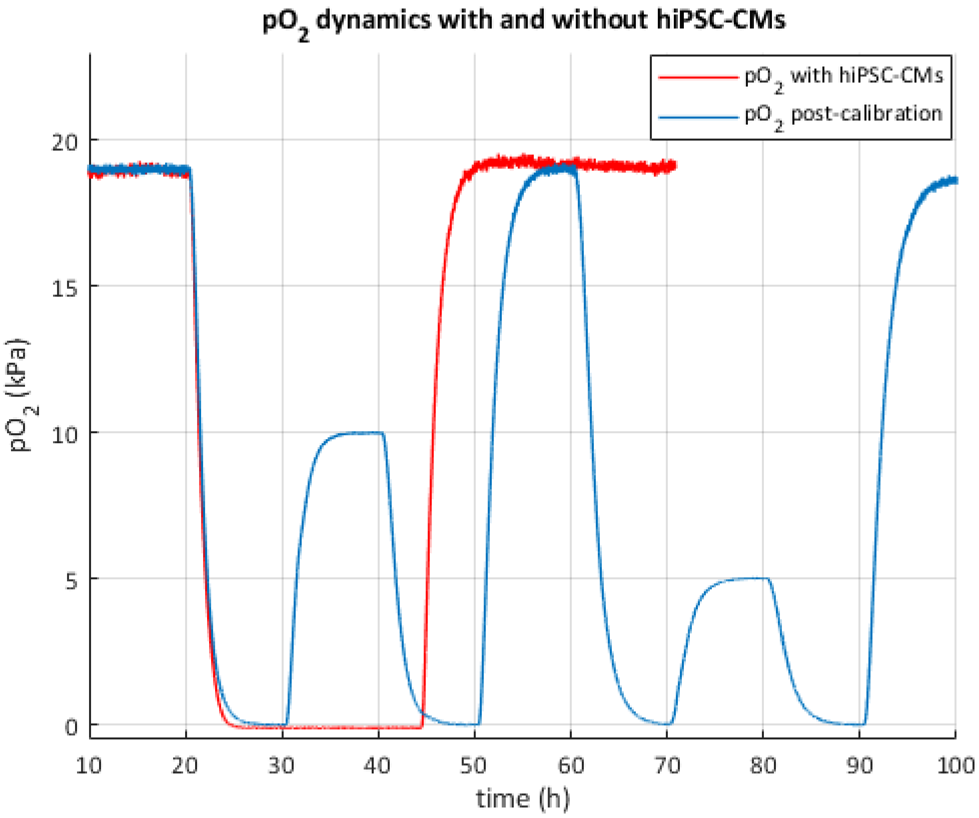

Supplementary Figure S 3. The post-calibration curve of the oxygen sensor (blue line). The sensor responses are measured at pO_2 values of 19 kPa, 10 kPa, 5 kPa and 0 kPa in the same conditions but without cells to calibrate the sensor. The same calibration values are used to

plot the pO_2 during the MEA mesurement with cells (red line). The curves show excellent functioning of the sensor. Small diffrences between the measurements with and with out cells can be seen at low pO_2 values. Evidently, due to the oxygen consumption of the cells, the lowest pO_2 is 0.1 kPa lower with cells than without and the hypoxia is stablized more rapidly with cells as well.

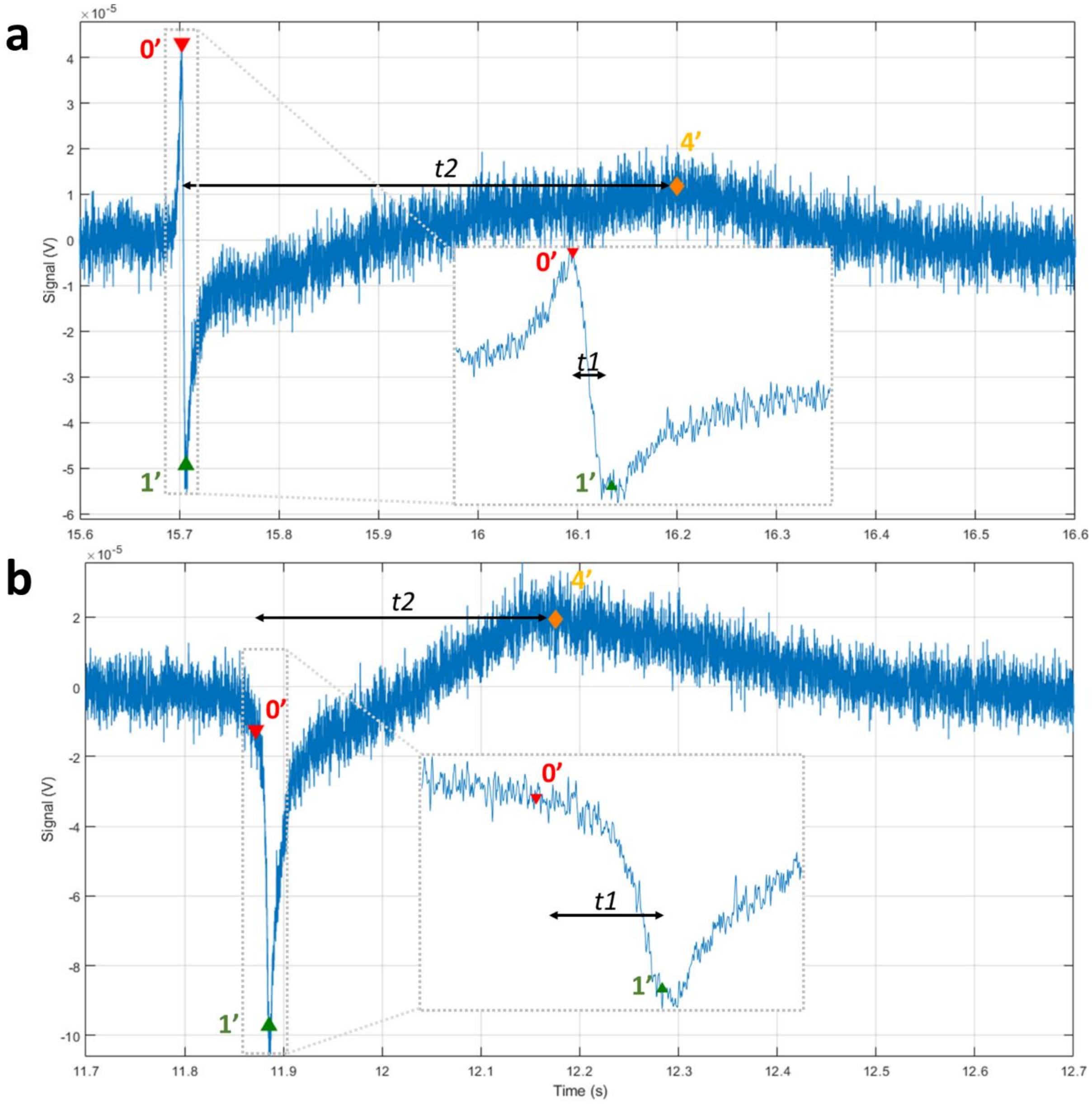

Supplementary Figure S 4. Depolarization time (t1) and field potential duration (t2) were determined from the microelectrode array field potential signals as shown in the panels a) and b) for two different types of signal forms. Depolarization time was determined as the time from 0' to 1', whereas field potential duration was determined as the time from 0' to 4'. The field potential phases 0', 1', 2', 3' and 4' as well as their relationship to action potential are described in a study by Tertoolen et al[1].

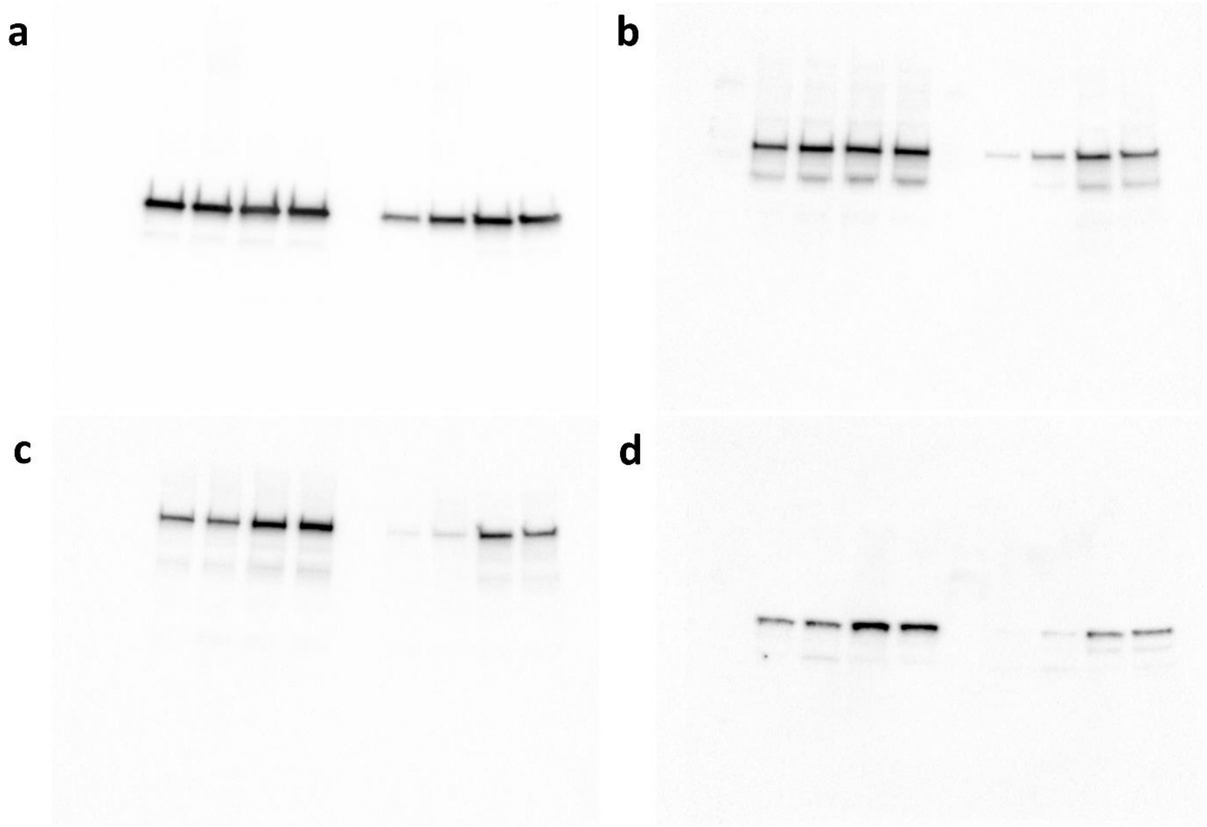

Supplementary Figure S 5. Blots from western blotting 8 h hypoxia and 24 h hypoxia protein samples. β-actin (A), HIF1α (B), MyBPC3 (C) and Troponin T (D) expressions in 8 h hypoxia, 8 h control, 24 h hypoxia and 24 h control samples. From left to right, there are 2 biological replicates of each condition for 8 h hypoxia, 8 h control, 24 h hypoxia and 24 h control samples.

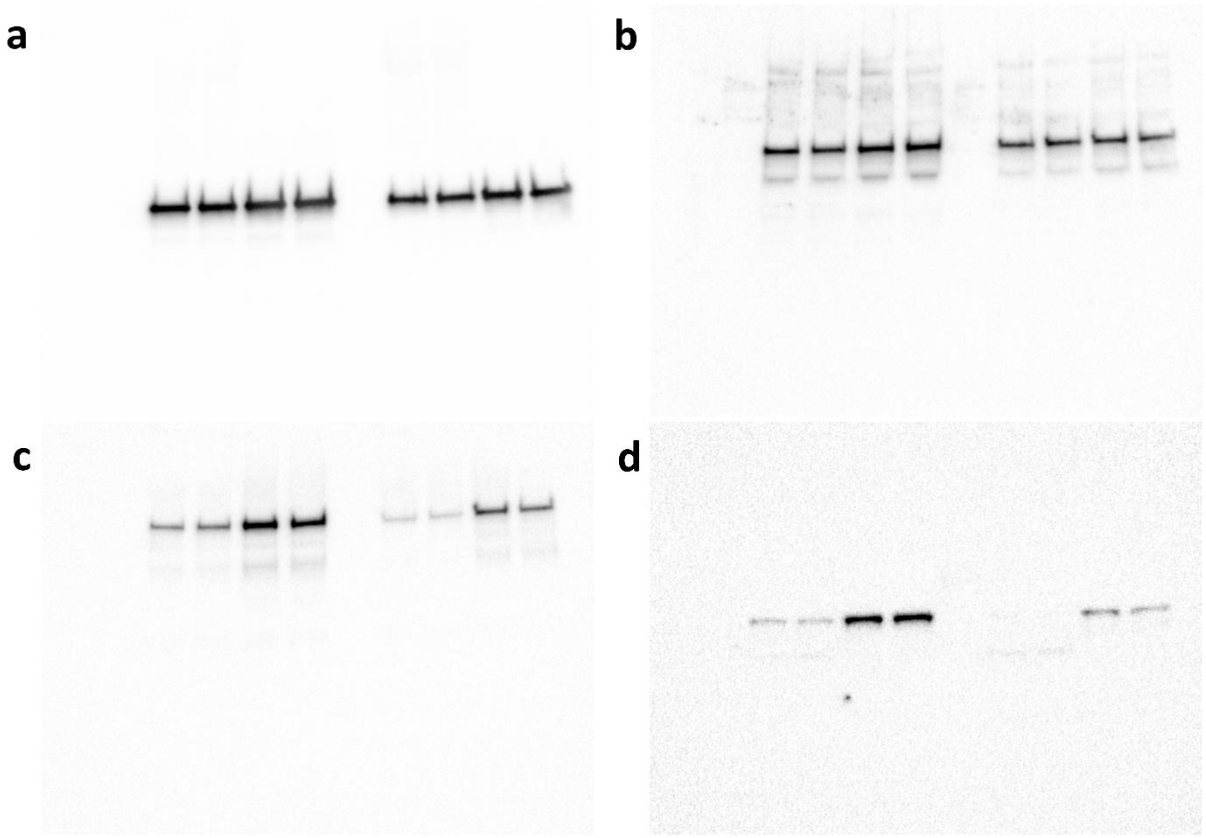

Supplementary Figure S 6. Blots from western blotting 8 h hypoxia-reoxygenation and 24 h hypoxia-reoxygenation protein samples. β-actin (A), HIF1α (B), MyBPC3 (C) and Troponin T (D) expressions in 8 h hypoxia-reoxygenation, 8 h control, 24 h hypoxia-reoxygenation and 24 h control samples. From left to right, there are 2 biological replicates of each condition for 8 h hypoxia-reoxygenation, 8 h control, 24 h hypoxia-reoxygenation and 24 h control samples.

References

1. Tertoolen, L. G. J., Braam, S. R., van Meer, B. J., Passier, R. & Mummery, C. L. Interpretation of field potentials measured on a multi electrode array in pharmacological toxicity screening on primary and human pluripotent stem cell-derived cardiomyocytes. *Biochem. Biophys. Res. Commun.* **497**, 1135–1141 (2018).
